# Implicit Mentalizing Persists beyond Early Childhood and Is Profoundly Impaired in Children with Autism Spectrum Condition

**DOI:** 10.3389/fpsyg.2016.01696

**Published:** 2016-10-28

**Authors:** Tobias Schuwerk, Irina Jarvers, Maria Vuori, Beate Sodian

**Affiliations:** ^1^Department of Psychology, Ludwig-Maximilians-UniversityMunich, Germany; ^2^Department of Psychiatry and Psychotherapy, University of RegensburgRegensburg, Germany; ^3^Institute of Medical Psychology, Ludwig-Maximilians-UniversityMunich, Germany

**Keywords:** mentalizing, implicit Theory of Mind, autism spectrum condition, executive function, eye tracking

## Abstract

Implicit mentalizing, a fast, unconscious and rigid way of processing other’s mental states has recently received much interest in typical social cognitive development in early childhood and in adults with autism spectrum condition (ASC). This research suggests that already infants implicitly mentalize, and that adults with ASC have a sustained implicit mentalizing deficit. Yet, we have only sparse empirical evidence on implicit mentalizing beyond early childhood, and deviations thereof in children with ASC. Here, we administered an implicit mentalizing eye tracking task to assess the sensitivity to false beliefs to a group of 8-year-old children with and without ASC, matched for chronological age, verbal and non-verbal IQ. As previous research suggested that presenting outcomes of belief-based actions leads to fast learning from experience and false belief-congruent looking behavior in adults with ASC, we were also interested in whether already children with ASC learn from such information. Our results provide support for a persistent implicit mentalizing ability in neurotypical development beyond early childhood. Further, they confirmed an implicit mentalizing deficit in children with ASC, even when they are closely matched to controls for explicit mentalizing skills. In contrast to previous findings with adults, no experience-based modulation of anticipatory looking was observed. It seems that children with ASC have not yet developed compensatory general purpose learning mechanisms. The observed intact explicit, but impaired implicit mentalizing in ASC, and correlation patterns between mentalizing tasks and executive function tasks, are in line with theories on two dissociable mentalizing systems.

## Introduction

Implicit mentalizing, or implicit Theory of Mind (ToM), a fast, unconscious and rigid way of processing others’ mental states, such as beliefs or desires, has recently received much interest in research on typical and atypical social cognitive development (Apperly and Butterfill, 2009; Baillargeon et al., 2010; Sodian, 2011; Senju, 2012). One key finding is that already children younger than 4 years of age implicitly mentalize. For example, results from violation of expectation paradigms suggest that already in their second year of life, children process an agent’s true and false belief. In these tasks, they look longer at events in which an agent acts incongruently to her mental state, e.g., when she does not look for an object where she believes it is located, but at a different location (Onishi and Baillargeon, 2005; Surian et al., 2007; Song et al., 2008). According to recent theories, implicit mentalizing continues to exist alongside the ability to explicitly mentalize, that is, to deliberately consider another’s mental state, in children above the age of 4 years, as well as in adults (cf., Apperly and Butterfill, 2009; Perner and Roessler, 2012).

Anticipatory looking paradigms revealed that children younger than 4 years of age not only expect an agent to act according to her beliefs, but that they also use her mental state to predict upcoming actions (Clements and Perner, 1994; Surian and Geraci, 2012; Thoermer et al., 2012; Low and Watts, 2013). In an influential study, Southgate et al. (2007) familiarized 25-month-olds with an agent and her goal to retrieve an object in one of two boxes. In the test trial, the agent was distracted and did not witness that the object was removed from the box she had lastly seen it in. When the agent turned back to the scene, the children’s anticipatory gaze indicated that they predicted the agent would open the door next to the now empty box. This suggests that they were sensitive to the agent’s false belief.

Another key finding is that individuals with autism spectrum condition (ASC) have an implicit mentalizing deficit (Senju et al., 2009; Schneider et al., 2013; Sodian et al., 2015). This deficit is thought to contribute to social interaction deficits in ASC (Frith, 2012; Senju, 2012). Employing equivalent anticipatory paradigms as described above, Senju et al. (2009) found that adults with ASC lacked the spontaneous appreciation of the agent’s false belief and did not reliably produce according action predictions.

Interestingly, this and other studies found a dissociation between implicit and explicit mentalizing (Senju et al., 2009; Schneider et al., 2013; cf., Abell et al., 2000): While implicit mentalizing seemed to be persistently impaired, participants with ASC were able to solve explicit mentalizing tasks. This led to the conclusion that individuals with ASC, especially those with high-functioning autism and Asperger syndrome, cope with explicit mentalizing deficits (Baron-Cohen et al., 1985) by developing compensatory strategies (Bowler, 1992; Happé, 1995; Ozonoff and Miller, 1995).

However, a recent study by Schuwerk et al. (2015) found that an implicit mentalizing deficit might be addressable by compensatory learning. In contrast to previous anticipatory looking false belief tasks, Schuwerk et al. (2015) presented the false belief-based action and its outcome, i.e., looking for the object in the empty box. Based on this perception-action contingency it is possible to form the non-mentalistic rule “if the agent did not witness the object transfer, she will look into the box that is empty by now.” Interestingly, after adults with ASC had the critical outcome information at the end of the first test trial, their performance on the second test trial in this implicit mentalizing task was comparable to that of neurotypical controls. This suggests that individuals with ASC might be able to modify their performance in an implicit mentalizing task by experience.

To date, we lack clear understanding of implicit mentalizing in typical and atypical social cognitive development in two aspects. First, we know little about an implicit mentalizing deficit in children with ASC. In the one study that documented this deficit in 8-year-old children with ASC, the children with and without ASC differed also to some extent in their explicit mentalizing competence (Senju et al., 2010). Thus, for drawing more firm conclusions about the implicit – explicit dissociation and impaired implicit mentalizing in ASC, one has to look at implicit mentalizing in children with and without ASC who pass explicit mentalizing tasks.

Moreover, a recent study employing a presumably more engaging mentalizing paradigm showed that 10-year-old children with ASC were able to spontaneously track another’s belief (Peterson et al., 2013). In a competitive game, one of two agents ended up with a false belief about the location of a prize. The other agent and the child knew where the prize was hidden. The child was encouraged to get the price, but only after he or she had nominated one of the agents to look for it first. Thus, to gain the price, the child should choose the agent with the false belief, who would not find it so that they could get it themselves. The majority of 10-year-olds with ASC opted for the agent with the false belief. At the same time, they performed poorly on a standard explicit false belief paradigm.

Although it is unclear to what extent the task by Peterson et al. (2013) tapped into the same implicit/spontaneous mentalizing ability as the anticipatory looking paradigm employed by Senju et al. (2010), their findings suggest that the conclusion that children with ASC have an implicit mentalizing deficit may be premature.

Second, we have only sparse empirical evidence on implicit mentalizing beyond early childhood, as most previous research studied infant or adult samples (for an example of the latter, see van der Wel et al., 2014). One recent example of a study investigating implicit and explicit mentalizing in older children comes from Grosse Wiesmann et al. (2016). These authors found the usual developmental trend in explicit false belief understanding between 3 and 4 years of age. However, implicit false belief understanding was already present and stable in this age range (see also Low, 2010). More evidence is necessary to conclude that implicit mentalizing continuously persists in parallel to a corresponding explicit system beyond early childhood, when children become increasingly proficient in advanced and second order mentalizing (e.g., Perner and Wimmer, 1985; Osterhaus et al., 2016).

To address these issues, we administered an implicit mentalizing task to a group of 8-year-old children with and without ASC, who were matched for chronological age, verbal and non-verbal intelligence, executive function skills^1^ and explicit mentalizing ability. We employed an anticipatory looking eye tracking paradigm that was previously used with adults (Schuwerk et al., 2015) and that was adapted from previous paradigms (Southgate et al., 2007; Senju et al., 2010). In this task, an agent looks for an object in one of two boxes. In two familiarization trials, she observes the self-propelled object entering one box, opens the box, and finds the object. In two test trials, the agent is distracted, and consequently ends up with a false belief about the object’s location and reaches for the object in the empty box.

We reasoned that if implicit mentalizing is a phenomenon dissociated from explicit mentalizing and specifically impaired in ASC, we should observe a group difference in the implicit mentalizing task in the current sample of children with ASC who are competent in explicit mentalizing tasks.

Further, as the previous study by Schuwerk et al. (2015) suggested that presenting the outcome of a false belief-based action leads to fast learning from experience and false belief-congruent looking behavior in adults with ASC, we were interested in whether already children with ASC learn from such information. If this were the case, we should find an effect of test trial repetition on false-belief congruent action anticipation in children with ASC.

## Materials and Methods

### Participants

A total of 14 children with ASC (*M*_age_ = 8.0 years, *SD* = 1.8 years; one female) participated in the study. Another 10 children with ASC were tested, but had to be excluded due to missing gaze data in one or both test trials of the implicit mentalizing task (*n* = 6) or not fulfilling inclusion criteria in the implicit mentalizing task (*n* = 4, see the data analysis section for details). All participants with ASC were clinically assessed by a psychologist or a psychiatrist and were required to fulfill the International Classification of Diseases-10th Revision (ICD)-10 criteria (American Psychiatric Association, 2013) for either Asperger Syndrome (*n* = 8), high-functioning autism (*n* = 5) or atypical autism (*n* = 1). We used two ASC screening tests which were filled by the caregivers to corroborate group assignment: the Social Responsiveness Scale (SRS), introduced by Constantino and Gruber, 2005 (German version by Bölte et al., 2008; cut-off criterion: *T*-score ≥ 60), and the Social Communication Questionnaire (SCQ, current form; discriminative cut-off: sum score ≥ 15) introduced by Rutter et al. (2003; German version by Bölte and Poustka, 2006). The SRS was used as a more general assessment of autistic traits whereas the SCQ questionnaire was applied as a measure of current communication skills and social functioning. In the SRS, the ASC group had a mean *T*-score of 80.8 (range = 70–100, *SD* = 9.1). The mean SCQ sum score was 16.6 (range = 9–27, *SD* = 6.0). Note that seven participants with ASC scored below the cut-off value of 15, indicating a currently alleviated communication skills and social functioning deficit in this subgroup.

The control group consisted of 21 neurotypical children (*M*_age_ = 7.2 years, *SD* = 1.4; six females). Five additional participants had to be excluded due to missing gaze data in one or both test trials of the implicit mentalizing task (*n* = 1) or not fulfilling inclusion criteria in the implicit mentalizing task (*n* = 4). The control group was matched for chronological age, *t*(31) = 1.45, *p* = 0.156, Cohen’s *d* = 0.52, non-verbal IQ, *t*(31) = -0.02, *p* = 0.988, Cohen’s *d* = -0.01, verbal IQ, *t*(31) = 1.26, *p* = 0.219, Cohen’s *d* = 0.45, and explicit ToM ability, *t*(31) = -1.64, *p* = 0.112, Cohen’s *d* = -0.59, based on the ToM scales by Wellman and Liu (2004, for details see the tasks and material section). The verbal and non-verbal IQ were obtained using subtests of the Wechsler Preschool and Primary Scale of Intelligence-III (WPPSI-III; Wechsler, 2002; German Version: Hannover-Wechsler-Intelligenztest für das Vorschulalter – III, HAWIVA-III; Ricken et al., 2007) and the Wechsler Intelligence Score for Children-IV (WISC-IV; Wechsler, 2004; German Version: Hamburg-Wechsler-Intelligenztest für Kinder – IV, HAWIK-IV; Petermann and Petermann, 2007). For the verbal IQ the subtest used from the WPPSI-III was Vocabulary (passive and active) and from the WISC-IV Vocabulary and Picture Concepts. For the non-verbal IQ the subtest Block Design and Matrix Reasoning were used from both IQ test. The control group had significantly less autistic traits as assessed by the SRS and SCQ. The average SRS *T*-score was 38.9 (range = 25–55, *SD* = 8.5), the average SCQ sum score was 2.7 (range = 0–7, *SD* = 1.7). There was a significant difference between the ASC group and the control group in both the SRS, *t*(33) = 13.91, *p* < 0.001, Cohen’s *d* = 4.84, and the SCQ, *t*(33) = 10.11, *p* < 0.001, Cohen’s *d* = 3.52.

The caregivers gave informed written consent. Children received a present for their participation. Their caregivers received monetary compensation for travel expenses. The ethics committee of the Department of Psychology and Education of LMU Munich approved the study.

### Tasks and Material

#### Implicit Mentalizing Task

**FIGURE 1:**
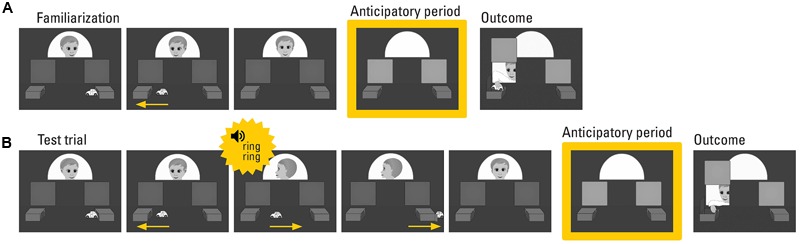
**Implicit mentalizing task: Still frames depicting key events of **(A)** one of two familiarization trials and **(B)** one of two test trials.** In the familiarization trials, the agent watches a car moving from one box into the other. She disappears behind the occluder, opens the door next to the box the car is in, and takes the car. In the test trials, the agent did not witness that the car left the box she had seen it disappear in and she fruitlessly looks for it in the empty box. Gaze data from the 3 s-long anticipatory period was analyzed. Each trial ended with the presentation of the belief-congruent action and its outcome.

This implicit mentalizing task was adapted from previous eye tracking false belief paradigms (Southgate et al., 2007; Senju et al., 2010; Thoermer et al., 2012). The same task was previously used with a sample of adults with and without ASC (Schuwerk et al., 2015). **Figure 1** provides an overview of trials and scene setup. In two familiarization trials (each lasting for 32 s), an agent watched a toy car moving from one into another box. Subsequently, the agent disappeared behind a screen. Two doors, one next to each box, were illuminated, accompanied by a chime. This scene was frozen for 3 s and served as anticipatory period. After that, the agent opened the door next to the box she had seen the toy car disappear in. Finally, she reached for the car. These two trials served to familiarize the participants that the agent wants to get the car and therefore opens one of the two doors. Second, participants learned about the contingency between the illumination of the doors/chime and the opening of the door in these familiarization trials. The subsequently presented two test trials lasted for 41 s each. In the test trials, the agent was distracted by a phone ring and did not see that the car, after arriving at the second box, drove back to the first box and then left the scene. Then the phone ringing stopped, the agent turned back to the scene, and disappeared behind the screen. The doors were illuminated, the chime sounded, and the 3 s-long anticipatory period started. Finally, the agent opened the door and reached for the box she falsely believed the car would be located in. Half of the participants watched horizontally flipped movies to counterbalance for the laterality of events.

#### Explicit Mentalizing Task

We used the ToM scale by Wellman and Liu (2004) to assess explicit mentalizing ability. The ToM scale consists of 6 tasks including the following concepts: diverse desires, diverse beliefs, knowledge access, contents false belief, real apparent emotion, and explicit false belief. We used the validated German version by Kristen et al. (2006). All tasks were presented with the help of toys and pictures. The first two tasks do not require a representational understanding of mental states whereas the following tasks do. Overall, a score of 6 for solving all six tasks could be achieved. We adhered to the procedure as described by Wellman and Liu (2004).

#### Executive Function Tasks

Executive function was assessed employing two card-sorting tasks, which draw on cognitive flexibility and self-control, namely the dimensional change card sorting task (DCCS) and the reversal shift test. Both tasks consist of two different sets of cards, which include two goal cards and 30 test cards. The goal cards were assigned to a box each and the test cards had to be sorted into the boxes according to a certain rule. There were always three phases for the tasks: a pre-switch phase, a post-switch phase, and a mix-phase that consisted of a mix of the previous two phases.

The DCCS (modified by Kloo et al., 2008) was administered according to the procedure described by Zelazo (2006). The two goal cards depicted a green apple and a red banana. Children were asked to sort cards either according to the form or according to the color. In the pre-switch phase, children had to sort everything according to color. In the post-switch phase, the rule was to sort according to form. The last phase, namely the mix-phase, was added to the procedure by Zelazo (2006), to have an additional level of difficulty and thereby the ability to further differentiate performance. In this last phase participants had to switch back and forth between the previous two tasks and rules.

The reversal shift test was based on the one-dimensional card sorting task by Perner and Lang (2002). The two goal cards showed an elephant and a bunny. Both cards had the color beige and therefore only differed in the type of animal shown. Here, the pre-switch phase was to play the game “correctly”, i.e., put the elephants to the elephants, etc., and the post-switch phase required playing the game “incorrectly”, i.e., putting the elephants to the bunnies and bunnies to the elephants. In the mix-phase, which was added just like in the DCCS, the rules were intermixed.

### Procedure

The children performed the implicit mentalizing task first. Eye tracking stimuli were presented with Tobii Studio (Version 2.2, Tobii Technology) on a Tobii T60 eye tracker (60 Hz sampling rate, inbuilt 17-inch TFT screen, 1280 × 1024 pixels; Tobii Technology, Stockholm, Sweden). The participants sat on a chair with a distance of approximately 60 cm from the screen. A 5-point calibration procedure preceded the stimulus presentation. The explicit mentalizing task and the executive function tasks were performed at a table with the experimenter seated across from the child. Subsequently, verbal and non-verbal IQ subtests were administered. Caregivers filled the SRS and SCQ questionnaires during the experimental session with the child.

### Data Analysis

Statistical analyses were conducted using IBM SPSS Statistics 23 (SPSS Inc., Chicago, IL, USA). As preliminary analyses revealed no influence of sex, data was collapsed across this variable. The significance level was *p* ≤ 0.05.

#### Implicit Mentalizing Task

Analyses of raw data were conducted using customized scripts in R (R Core Team, 2013). A velocity-based fixation filter (Salvucci and Goldberg, 2000) with a velocity threshold of 0.05°/ms was used to define the fixations. Additionally, a temporal threshold was set to exclude fixations that lasted less than 80 ms. As fixations on the doors during the 3 s-long anticipatory period were the critical measure, the two doors were chosen as areas of interest (AOIs; approximately 2.8° × 2.8°) for data analysis. The door the character opened after the anticipatory period was defined as the “correct door,” whereas the other door is referred to as the “incorrect door.” A differential looking score (DLS) according to Senju et al. (2009) was calculated by subtracting the total duration of fixations on the incorrect door from the total duration of fixations on the correct door, and then dividing it by the sum of the total duration of fixations on both doors. The DLS ranges from 1 (visual preference for correct door) to -1 (visual preference for incorrect door). A value around 0 indicates no preference for one of the two doors. Participants who had a looking bias toward the correct door in at least one of the two familiarization trials were included in the further analysis. Four children from the ASD group and four children from the control group had to be excluded as they did not show this belief-congruent anticipatory looking behavior. Note that this differs slightly from Senju et al. (2010), who only included participants who looked longer to the correct than to the incorrect door in the last familiarization trial. Senju et al. (2010) presented four familiarization trials in contrast to only two in the current study. This presumably made it easier for their participants to learn the contingency between the door illumination and the reaching action. Because of this, and to be consistent with our previous study with adults, we adjusted our criterion. Applying Senju et al.’s criterion to our sample would have resulted in excluding five additional participants with ASC and one additional control participant. Notably, preliminary analyses revealed that using Senju et al.’s criterion did not change the pattern of DLS results.

Additionally, first looks toward the two doors in the anticipatory period were analyzed. It was coded whether the first fixation after the illumination of the doors was on the correct or incorrect door.

#### Explicit Mentalizing Task

To pass each of the ToM scale subtests it was required to answer both the test and the control questions correctly. For each solved task a point was given resulting in a maximum of 6 points. The percentage of correct responses was used for statistical analyses. One child with ASC and one child from the control group refused to take part in the ToM scale. A second coder recoded test and control questions of 33% of the whole sample from a video recording of the test session The Inter-rater reliability revealed an agreement of 100% (Cohen’s kappa = 1).

#### Executive Functions Task

To pass the DCCS and the reversal shift tasks a certain number of cards had to be sorted correctly. For the first two phases of the tasks it was necessary to sort at least five cards correctly. To pass the last phase, at least nine cards had to be sorted correctly (Zelazo, 2006). The third phase was only administered if a child sorted at least five cards in each of the other phases correctly. None of the children failed the pre-switch-phase. One control child refused to take part in the executive function tasks. The maximum that could be achieved in this set of tasks was a score of 6 for passing all three phases of both tasks. Statistical analyses are based on the percentage of correct responses. Inter-rater reliability was assessed as in the explicit mentalizing task. It again revealed a perfect agreement (Cohen’s kappa = 1).

## Results

### Implicit Mentalizing Task

**FIGURE 2:**
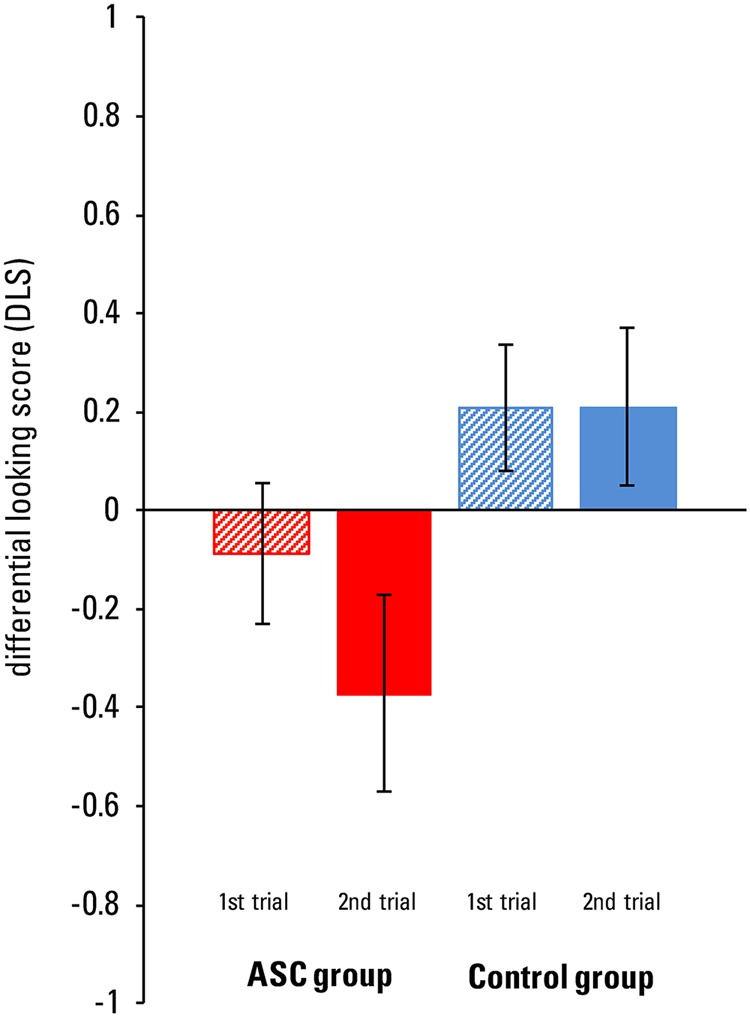
**Implicit mentalizing task: mean differential looking scores (DLS; ±SEM) from children with ASC and children from the control group per test trial**.

The DLS was analyzed via a 2 × 2 repeated measures ANOVA with the between factor group (ASC group, control group) and the within factor test trial (first, second). **Figure 2** displays mean DLS scores for group and test trial. The ANOVA revealed a significant effect of group, *F*(1,33) = 10.55, *p* = 0.003, η^2^ = 0.24, but no effect of test trial, *F*(1,33) = 0.60, *p* = 0.446, η^2^ = 0.02. There was also no significant interaction between group and test trial, *F*(1,33) = 0.34, *p* = 0.441, η^2^ = 0.02. Overall, the control group showed a stronger looking bias toward the correct door (*M* = 0.21, *SD* = 0.42) compared to the ASC group (*M* = -0.23, *SD* = 0.33).

Because of our *a priori* interest in a potential learning effect from one test trial to another, we checked for significant differences in DLS scores between the first and second test trial within each group. Neither in children with ASC, nor in the control group, the DLS differed between the first and second test trial [ASC group: *t*(13) = 0.96, *p* = 0.357, Cohen’s *d* = 0.53; control group: *t*(20) = -0.01, *p* = 0.995, Cohen’s *d* = 0.00]. Consequently, we collapsed the DLS score across both test trials for further analyses.

To check whether children from the ASC group and the control group had a looking bias significantly different from chance, we calculated one-sample *t-*tests against zero for each group. The control group looked significantly more at the correct door compared to the incorrect door, *t*(20) = 2.28, *p* = 0.034, Cohen’s *d* = 1.02, whereas the ASC group looked significantly more at the incorrect door compared to the correct door, *t*(13) = -2.53, *p* = 0.025, Cohen’s *d* = -1.41.

**FIGURE 3:**
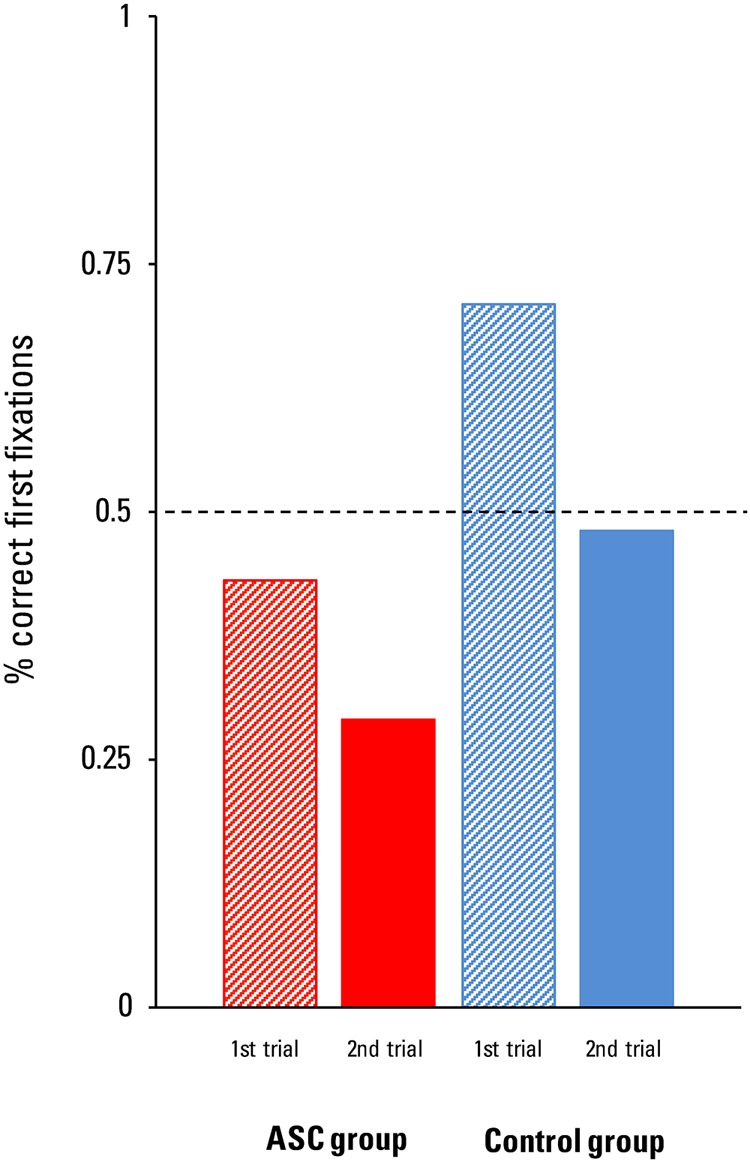
**Implicit mentalizing task: percentage of correct first fixations from children with ASC and children from the control group per test trial.** The dotted line indicates chance level.

For the first fixations, a binominal logistic regression was calculated with the dichotomous dependent variable performance (0 or 1) and the categorical independent variables group (ASC group = 1, control group = 0) and test trial (first test trial = 0, second test trial = 1). **Figure 3** shows percentage of correct first fixations per group and test trial. The intention was to assess the influence of group and test trial repetition on the location of first fixations. The logistic regression model was statistically significant, χ^2^(1) = 6.84, *p* = 0.033; and explained 12.4% (Nagelkerke *R*^2^) of the variance in first fixations. The model correctly classified 62.9% of all cases. There was a significant effect of group as a predictor (*B* = 1.02, *SE* = 0.52, *Wald* = 3.86, *p* = 0.049). Participants with ASC were 2.77 times more likely to direct their first fixation to the incorrect door as compared to the controls. There was no significant effect of test trial as predictor (*B* = 0.86, *SE* = 0.51, *Wald* = 2.91, *p* = 0.088).

Analogous to the DLS analysis, we compared first fixations between the first and second test trial within each group. There was neither a difference in first fixations between trials for the ASC group (*p* = 0.414, McNemar’s Test, one-tailed), nor for the control group (*p* = 0.687, McNemar’s Test, one-tailed).

To check whether children from the ASC group and the control group had a first fixation preference for the correct door significantly different from chance, we created a percentage score over both test trials, which was then tested against zero for each group. Neither the ASC group, *t*(13) = -1.47, *p* = 0.165, Cohen’s *d* = -0.82, nor the control group differed significantly from chance, *t*(20) = 1.45, *p* = 0.162, Cohen’s *d* = 0.65.

### Explicit Mentalizing Task

The control children achieved an average performance of 85% (range = 25–100%, *SD* = 21%). The children with ASC solved 70% (range = 25–100%, *SD* = 31%) of tasks from the ToM scale. Performance of children with ASC and controls did not significantly differ, *t*(31) = -1.51, *p* = 0.112, Cohen’s *d* = -0.54.

### Executive Function Task

In the executive function tasks the control children solved an average of 98.3% of the tasks correctly (range = 5-6, *SD* = 6.6%). The ASC group solved 81.7 tasks on average (range = 2–6, *SD* = 21.7%). The groups differed significantly in their executive function skill, *t*(32) = -3.26, *p* = 0.003, Cohen’s *d* = -0.99.

### *Post hoc* Analyses

As the two groups differed in executive function, the ANOVA of the DLS performance - the variable of key interest - was repeated with executive function task performance as a covariate. The pattern of results did not change. We again found a significant effect of group, *F*(1,31) = 5.57, *p* = 0.025, η^2^ = 0.15, but no effect of test trial, *F*(1,31) = 0.23, *p* = 0.634, η^2^ = 0.01. Also the group × test trial interaction was not significant, *F*(1,31) = 0.28, *p* = 0.471, η^2^ = 0.02. Additionally, we checked for each group whether executive function tasks performance is related to performance in the implicit and explicit mentalizing task. Neither in children with ASC, nor in the control group, a significant correlation between performance in the implicit mentalizing task and the executive function tasks was observed (ASC group: *r* = 0.34, *p* = 0.232; control group: *r* = 0.36, *p* = 0.879). However, there was a significant correlation between executive function tasks performance and explicit mentalizing task performance in the ASC group (*r* = 0.61, *p* = 0.012) and the control group (*r* = 0.45, *p* = 0.045).

## Discussion

The current implicit mentalizing task revealed a difference between 8-year-old children with ASC and matched control children in the spontaneous anticipation of an agent’s false belief-based action. Whereas neurotypical children’s looking bias over two test trials suggests that they predicted the agent’s action based on her false belief, children with ASC lacked this appreciation of the agent’s false belief-congruent action. In contrast, over both test trials, they even displayed a significant looking bias toward the incorrect door. Repeating the test trial had no effect on anticipatory looking.

The finding that 8-year-olds with ASC did not systematically generate false belief-based action anticipations confirms an implicit mentalizing deficit in children with ASC, previously documented in 10-year-olds (Senju et al., 2010). However, in the previous study by Senju et al. (2010), children with ASC and their controls differed not only in implicit, but also in explicit mentalizing task performance. Thus, it could not be ruled out completely that group differences in implicit mentalizing arose from differences in explicit mentalizing, maybe mediated by verbal intelligence. Our results advance previous research by showing that poor performance persists, even when children with ASC and their control group are closely matched for chronological age, verbal and non-verbal intelligence and explicit mentalizing skills. Together, both studies point to a specific deficit in implicit mentalizing in children with ASC.

By repeating the test trial, we were able to check whether participants learned from the presentation of the false belief-based action and its outcome. The lacking effect of test trial repetition indicated no experience-based modulation of anticipatory looking in the repeated presentation of the test trial. Consequently, we collapsed gaze data over the two test trials for each group. Comparing anticipatory looking over both test trials against chance performance showed that neurotypical children systematically predicted that the agent would open the box in which she falsely believed the car would be located. This finding helps closing a gap of evidence on implicit mentalizing beyond early childhood. Recent two-systems accounts on mentalizing claimed that implicit mentalizing is already present in infancy and co-exists in parallel to later developing explicit mentalizing (Apperly and Butterfill, 2009). Yet, this remains to be proven empirically. Together with other recent work (Low, 2010; Senju et al., 2010; Grosse Wiesmann et al., 2016), our findings suggest that implicit mentalizing indeed is a phenomenon presumably persisting across lifespan.

Contrary to what we expected, test trial repetition had no effect on anticipatory looking in 8-year-old children with ASC. In a previous study with adults with ASC, showing the false-belief based action and its outcome (i.e., the agent opens the empty box and vainly looks for the ball) only once, was sufficient to increased the looking bias toward the false belief-congruent door in the second test trial (Schuwerk et al., 2015). In the second test trial, adults with ASC performed as good as neurotypical controls. This suggested that rapid learning from action-outcome contingencies modulated gaze behavior in this implicit mentalizing task. However, although the current stimulus material was identical, this was not what we observed in the present sample of children with ASC. On the contrary, in the second test trial the DLS was even more negative than the one in the first test trial, what led to a significant looking bias toward the incorrect box.

A possible explanation for this finding is the counterbalancing of our stimulus material across the two test trials. In the first test trial, the agent opened the right door (left door, respectively) to look for the car. In the second test trial, the presented movie was flipped horizontally, so that the door that would be opened was on the opposite side. It could be that children with ASC perseverated on the location in which they saw the agent reaching for the car in the previous test trial. This could in turn reflect a simple action prediction strategy, which might be fruitful in several cases, but not in the current situation. It seems that children with ASC let themselves be guided by superficial scene properties (i.e., location) and that they were not yet able to use action-effect contingencies. Yet, future research is needed to pin down whether children with ASC make use of such a location-bound action prediction strategy.

In summary, our findings point to a sustained implicit mentalizing deficit that cannot be easily addressed by experience. It seems that 8-year-old children with ASC, in contrast to adults with ASC, are not yet capable of employing information about perception-action contingencies to compensate for an implicit mentalizing deficit.

Notably, our group of children with ASC performed poorer than the control children in the executive function tasks. To check whether poorer executive function could contribute to the observed group difference in the implicit mentalizing task, we ran *post hoc* analyses. First, including the performance in the executive function tasks as a covariate and the DLS analysis revealed the same pattern of results. Second, within each group, executive function performance was unrelated to anticipatory looking in the implicit mentalizing task. This gives us good reason to conclude that anticipatory looking in the current implicit mentalizing task did not rely on voluntary cognitive control and that the lacking systematic false belief-based action prediction of children with ASC cannot be explained by poorer executive function skills.

Interestingly, our *post hoc* analyses revealed a positive correlation between performance in the executive function tasks and explicit mentalizing ability. This is in line with a large body of evidence on the close link between both cognitive domains (for a recent meta-analysis, see Devine and Hughes, 2014). Further, consistent with recent evidence (Low, 2010; Grosse Wiesmann et al., 2016), we found that executive function task performance was related to performance in the explicit, but not in the implicit mentalizing task. This provides further support for two-systems accounts of mentalizing (Apperly and Butterfill, 2009; cf., Perner and Roessler, 2012). The implicit system enables already young children to be sensitive to false beliefs. This system works involuntarily, fast, effortless, but inflexibly. The around the age of 4 developing explicit system allows to voluntary switch perspectives and to consider another’s false belief to generate action explanations. This system is flexible but slow, and draws on cognitive resources.

When investigating social cognition in ASC using eye tracking, potentially confounding deficits in general visual processing have to be taken into account. In other words, is the group difference we observed in the implicit mentalizing task attributable to an implicit mentalizing deficit, or did this difference arise from general – and not specifically social – visual processing deficits in ASC? The following aspects of our paradigm help to address alternative explanations in terms of general atypical visual processing in the group of children with ASC. First, to account for a potentially weaker saccadic accuracy in ASC (e.g., Schmitt et al., 2014), a calibration procedure prior to the implicit mentalizing task ensured that the fixation of targets was sufficiently accurate. Second, Wang et al. (2015) recently reported atypical visual saliency in the first few seconds of scene perception in ASC. The scene, the agent and the objects of the present paradigm, were introduced for several minutes to avoid potential group differences in early visual processing of the scene. Third, events took place slowly, and the anticipatory period was statistic without displaying an agent. This should render any impact of movement/biological motion and social stimuli processing deficits in ASC (Blake et al., 2003; Dakin and Frith, 2005; Guillon et al., 2014) neglectable.

Future research that carefully contrasts social and non-social stimuli is necessary to unravel the relationship between implicit social cognitive and rather general visual processing characteristics in ASC (for an example, see von Hofsten et al., 2009). To date, it is unclear whether these two are independent phenomena, whether visual processing characteristics contribute to social cognitive deficits (Behrmann et al., 2006; Hellendoorn et al., 2014), or whether both are manifestations of an impaired underlying cognitive ability (Sinha et al., 2014).

In summary, our findings provide support for a persistent implicit mentalizing ability in neurotypical development beyond early childhood. The observed intact explicit, but impaired implicit mentalizing in ASC, and the observed link between executive functions and explicit, but not implicit mentalizing, is in line with theories on two dissociable mentalizing systems. Further, it seems that 8-year-old children with ASC are not yet capable of employing information about perception-action contingencies to compensate for an implicit mentalizing deficit.

## Author Contributions

Conceptualization, MV and BS; Methodology, MV and BS; Formal Analysis, TS, MV, and IJ; Investigation, MV; Resources, BS; Writing-Original Draft, TS, and IJ; Writing-Review and Editing, TS, IJ, MV, and BS; Visualization, TS; Supervision, BS; Funding Acquisition, BS.

## Conflict of Interest Statement

The authors declare that the research was conducted in the absence of any commercial or financial relationships that could be construed as a potential conflict of interest.

## Footnotes

1 The two groups were meant to be matched for executive function. However, although both children with ASC and controls performed comparably well, they differed significantly in their executive function skill. We provide details on this result, *post hoc* analyses and a discussion of this finding.
